# Evaluation of patients’ knowledge on warfarin in outpatient pharmacy of a tertiary care cardiac center

**DOI:** 10.1186/s13104-015-1416-1

**Published:** 2015-09-10

**Authors:** Sweta Shrestha, Binaya Sapkota, Anjana Kumpakha, Upasana Acharya, Rashmi Sharma

**Affiliations:** Kathmandu University School of Science Department of Pharmacy, Kathmandu, Nepal; Government of Nepal Civil Service Hospital, Minbhawan, Kathmandu, Nepal; Karnali College of Health Sciences, Kathmandu, Nepal

**Keywords:** Warfarin, Anticoagulation knowledge assessment, Adverse drug reaction, Narrow therapeutic index

## Abstract

**Background:**

Warfarin is widely used for the prevention and treatment of cardiac, thromboembolic and hypercoagulable diseases. Since warfarin is a narrow therapeutic index medicine, it requires close monitoring when used in the outpatient setting and on long term basis. Warfarin has been found to be associated with a number of complications especially bleeding. Patients’ knowledge on warfarin can improve anticoagulation control with decrease in adverse drug reaction and other associated complications. The objective was to assess knowledge level of warfarin therapy among its users and to provide adequate education and counseling to the patients.

**Methods:**

In the present cross sectional study, 34 patients on warfarin were interviewed. Patients’ knowledge on warfarin was assessed using a validated Anticoagulation Knowledge Assessment (AKA) questionnaire comprising 29 questions. Each correct answer scored 3.45 points whereas an incorrect answer scored zero point. Patient who answered at least 21 questions correctly or scored (21 × 3.45 = 72.4 %) was considered to have adequate level of knowledge or have obtained a passing score. Association between independent variables and AKA score was assessed using Pearson Chi square test or Fisher’s exact test for categorical variables. Patients were counseled regarding proper warfarin use by the researcher pharmacists at the end of each data collection schedule.

**Results:**

Of the 34 patients, only 5.8 % (n = 2) achieved a passing score whereas 94.1 % (n = 32) failed to achieve the passing score. 67.6 % of the patients (n = 23) achieved a score below 50 %. More than 50 % of the patients incorrectly answered 15 questions in the questionnaire. None of the patients scored 100 %. No significant association was found between age, gender of patients and total warfarin score. A significant association (p < 0.05) was found between duration of warfarin therapy and total warfarin score.

**Conclusions:**

Warfarin knowledge was poor among the patients. Hence, regular counseling with timely assessment of their understanding was felt necessary.

## Background

Warfarin is the preferred oral anticoagulant in conditions requiring prolonged anticoagulation [1–4]. It is widely used for the prevention and treatment of cardiac, thromboembolic and hypercoagulable diseases [1, 2, 5–8]. Since warfarin is a narrow therapeutic index medicine, it requires close monitoring when used in the outpatient setting and on long term basis [2, 3, 5, 9]. Warfarin has been found to be associated with a number of complications especially bleeding [10, 11]. Moreover, among the list of prescription medications it has been listed as a highly interacting drug with food and other medications [2, 11] and consequently improper control over anticoagulation can either result into compromised drug efficacy or increased risk of haemorrhage [2, 11–13]. Hence, monitoring of the International Normalized Ratio (INR) of the patients on warfarin becomes very essential to create a balance between its anticoagulation efficacy and possible bleeding complications [4, 5]. Knowledge of warfarin’s action, its potential side effects, interacting drugs and food, cautions to be taken and significance of adherence to warfarin plays an important role in achieving the desired therapeutic outcome while preventing the adverse reactions [2, 13, 14].

Providing adequate education on warfarin with the aim of improving patients’ knowledge can be a useful strategy to decrease the possible adverse drug reactions and other associated complications. Proper knowledge on warfarin can also help patients attain a more stable anticoagulation control thus decreasing the frequency of INR checks and hospitalizations [3, 8, 15, 16]. A deficit in knowledge among patients taking warfarin has been shown especially in elderly which could be one of the important factors determining the degree of anticoagulation control [3, 4, 13, 15]. This makes it important to assess level of knowledge of warfarin therapy among its user and provide adequate education and counselling to the patients [1–4, 13, 15].

To date, none of the studies have assessed the knowledge level among warfarin users in Nepal. Realizing the need to fulfil this gap we conducted this study with the aim of determining the knowledge level among patients taking warfarin in Shahid Gangalal National Heart Centre, Nepal.

## Methods

### Study design and setting

This was a quantitative, cross sectional, single center study conducted over a period of 8 months in Shahid Gangalal National Heart Centre, Nepal. The major indications for warfarin therapy among the patients selected for the study were: Atrial fibrillation (AF), Deep vein thrombosis (DVT), Atrial fibrillation with valve replacement and Mitral stenosis with Atrial fibrillation. Target INR for most indications of warfarin was 2.5 (range 2.0–3.0).

### Study procedure

Patients above 18 years who visited the outpatient pharmacy and had been taking warfarin for at least 2 months were interviewed from August 2013 to March 2014. Patients who refused to participate in the study were excluded. A face to face interview was carried out using a pre-validated Anticoagulation Knowledge Assessment (AKA) questionnaire [16, 17]. Each question and its options were read to the patients by the researcher and the patient had to choose one among the four given options. Demographic information such as patients’ age, gender and other clinically relevant information such as duration of warfarin therapy and preferred source of counseling was also collected. A total of 34 patients were interviewed. After completion of the interview the patients were told the correct answer for each of the 29 questions in the questionnaire. Further counseling on warfarin therapy was also done.

### Ethical consideration

Shahid Gangalal National Heart Centre granted permission for the study to be conducted and approved ethically.

### Data collection instrument

The AKA questionnaire is a validated instrument designed to assess the patients’ anticoagulation knowledge [16, 17]. It comprises of 29 multiple choice questions. Each correct answer scored 3.45 points whereas an incorrect answer scored zero point. Patient who answered at least 21 questions correctly or scored (21 × 3.45 = 72.4 %) was considered to have adequate level of knowledge or have obtained a passing score [17].

### Statistical analysis

Anticoagulation knowledge level was determined based on total AKA questionnaire score and number of the questions answered correctly. Data were analyzed via statistical package for the social sciences (SPSS) version 22. Associations between the independent variables and the AKA score was assessed using Pearson Chi square test or Fisher’s exact test (when the individual cell frequency is less than 5) for categorical variables, considering the power of the study to be 80 % while selecting the sample size by applying simple random sampling technique.

## Results

Of the 34 patients enrolled in the study, the mean age was 60 ± 14.6. Of the study population 55.9 % was male. Most of the patients had been on warfarin therapy for less than 1 year (n = 19; 55.8 %) whereas 44.1 % of the study population (n = 15) were receiving warfarin therapy for more than 1 year. When inquired about the preferred source of counseling 91.1 % (n = 31) of the study population wished to receive counseling on warfarin therapy from the doctors whereas only 8.8 % (n = 3) preferred pharmacists as the counselors. Demographic data are presented in Table 1.

**Table 1:** Characteristics of study population

Characteristics	Frequency (%)
Gender	
Male	19 (55.8)
Female	15 (44.1)
Age group	
18–38	5 (14.7)
39–59	7 (20.5)
60–80	22 (64.7)
Preferred counselor	
Physicians	31 (91.1)
Pharmacists	3 (8.8)
Warfarin therapy duration	
2–12 months	19 (55.8)
>1 year	15 (44.1)

Of the 34 patients only 5.8 % (n = 2) achieved a passing score or were able to answer at least 21 questions correctly whereas majority of the study population i.e., 94.1 % (n = 32) failed to achieve the passing score. 67.6 % of the patients (n = 23) achieved a score below 50 %. Out of the 29 questions 15 questions were incorrectly answered by more than 50 % of the patients. None of the patients were able to score 100 %. Percentage of patients incorrectly answering the questions was presented in Table 2.

**Table 2:** Response to questions

Questions	Incorrect answer (percentage**)**
Non-steroidal drug choice	44.1
Food warfarin interaction	76.4
Warfarin effect leading to emergency unit	58.8
Managing missed dose	55.8
Spinach intake frequency	70.5
Interaction with alcohol	73.5
Action to take when buying multivitamin	23.5
Action to take when you run out of prescription	29.4
Most likely side effect of warfarin	67.6
Action to take when buying cold medication	61.7
Action to take when taking dental appointment	23.5
Action to take when taking antibiotic	58.8
Action of warfarin	79.4
Best time to take warfarin	14.7
Effect seen when INR is high	41.1
Drink decreasing warfarin effectiveness	94.1
Situation where visiting emergency is essential	61.7
Action in taking herbal supplements	67.6
Frequency of PT/INR test	44.1
PT/INR test result indicates	44.1
Side effect to be informed to Coumadin clinic	70.5
Test to be carried out routinely	50
Interaction of over-the-counter drugs with warfarin	47
Warfarin effect requiring emergency unit visit	44.1
warfarin food interaction	26.4
Action to take if you have both generic and brand of warfarin	44.1
Time warfarin stays in body system	82.3
Length of time warfarin needs to be taken	94.1
Most risky activity to avoid while on warfarin	32.3

No significant association was found between the age, gender of the patients and the total warfarin score, considering the power of the study to be 80 %. A significant association (p < 0.05) was found between duration of warfarin therapy and the total warfarin score.

## Discussions

Of the total study population 94.1 % was found to have inadequate knowledge on their warfarin therapy. This figure was found to be higher than the findings by Hu et al. (n = 100, 61 %), Baker et al. (n = 185, 74.1 %) and Nasser et al. [4, 17, 18]. This indicated poor knowledge level among the study population which might be either due to lack of proper understanding among the patients or due to lack of adequate and repetitive counselling.

Lowest score was obtained for patients’ knowledge on how long warfarin should have been taken once started and the drinks that could decrease the effectiveness of warfarin. This was in contrast to the findings of Khudair et al. who found the least level of knowledge was on warfarin drug interactions, effect of a missing dose and its management and whether or not healthcare professionals should have been informed on their current therapy [5].

Majority of the study population preferred physicians as their preferred counsellor on warfarin therapy (n = 31, 91.1 %). Similar findings have been reported by Khudair et al. who reported 57 % of the patients (n = 80) preferred physicians as their warfarin counsellor [5].

On average, 58 % of the study population were unaware of the interaction of warfarin with food while 48.3 % of the patients were unaware of the need of consulting the healthcare professionals prior to taking other medications. The review study by Nasser et al. had similar findings where 60–80 % of the patients were unaware of the drug/food interactions with warfarin [4]. Of the study population 44.1 % of the patients were unaware of the interaction of alcohol with warfarin. This finding was consistent with the result obtained by Roche et al. and another study by Yahaya et al. where 22–49 % of the patients and 34.6 % of the study population respectively were unaware of the interaction of alcohol and warfarin [2, 3]. In regard to the need of avoidance of non-steroidal anti-inflammatory drugs, 44.1 % of the study population lacked proper understanding similar to the findings in the survey by Campbell and in a study by Roche where 27 % of the patients were unaware of the need to avoid aspirin [3, 14].

In our study no association was found between age and knowledge score of the patients, considering the power of the study to be 80 %. The findings were in contrary to the findings in studies where age was found to have negative effect on total knowledge score of the patients [4, 15, 18, 19]. Similarly, no association was found between gender and the total knowledge score which was supported by previous studies [2, 5, 15, 18]. Knowledge was related to duration of therapy. Similar findings were presented by Tang et al. but the finding was in contrast to the result obtained by Hasan et al. where no association was found between duration of therapy and knowledge [10, 15].

In a study by Imran F. Khudair in Hamad General Hospital, Doha, the author reported Physicians to be the main educators who may not have sufficient time to provide proper counseling. Also, the author reported that most patients preferred to receive counseling on warfarin from the physicians followed by pharmacists and then the nurses. In our study we wanted to know the concept of the general public as to whom they perceive as the main educator on warfarin.

### Study limitation

Altogether 175 patients were asked to participate in the study, out of which only 34 patients (19.43 %) agreed for participation. Sample size was small to generalize the study. Large scale and multi-centric study would be mandatory to generalize the study findings, for which this study could become a pathfinder in a resource constraint country like Nepal.

## Conclusions

The findings of this study show that majority of the patients have poor level of knowledge on warfarin. Age and gender of the patients were found to have no correlation with the knowledge score whereas a significant association was found between duration of warfarin therapy and the total knowledge score. Patients should have proper knowledge on the adverse effects, mechanism of action, dietary and drug interactions of warfarin and actions to be taken if adverse effects are seen or in case of missed dose. The gap in knowledge level of patients can be filled by providing education to the patients and the healthcare providers play an important role in it.

## Abbreviations

AKA: anticoagulation knowledge assessment
INR: International Normalized Ratio
